# Stab Wound of Percardium

**Published:** 1916-05

**Authors:** 


**Stab Wound of Percardium.** Dr. Fred Rankin, ibid. reports a case of self-inflicted scissor wound in a man of 35, first seen half an hour after the injury, there being no shock. On account of dyspnoea, the stomach tube was passed but only a small amount of clear fluid withdrawn. Two hours later, it was decided to operate. There was much haemorrhage so that the internal mammary artery was tied. The scissors had penetrated the seventh cartilage and opened the pericardium, from which large clots were removed. Suture was not attempted. The heart was apparently intact but the condition of the patient at the time did not warrant protracted search. The wound was packed, after resecting ribs, without suture and pus developed but the general condition was good till the ninth day when death suddenly occurred. No autopsy, tentative diagnosis of embolism. The first deliberate attempt at surgery of the heart was in 1871 when Callender removed a needle. Roswell Park in 1877 aspirated a myocardial abscess. Farina, 1896, first introduced sutures for a wound of the heart.

